# A review of the occurrence, distribution, and impact of bitumen seeps on soil and groundwater in parts of southwestern Nigeria

**DOI:** 10.1007/s10661-023-10960-0

**Published:** 2023-02-01

**Authors:** Solomon Mayowa Jekayinfa, Michael Adeyinka Oladunjoye, Kennedy O. Doro

**Affiliations:** 1 Geoscience Department, Pan-African University of Life and Earth Sciences, Ibadan, Nigeria; 2 Department of Geology, University of Ibadan, Oduduwa Road, 200132 Ibadan, Nigeria; 3 Department of Environmental Sciences, The University of Toledo, 2801 West Bancroft Street, Toledo, OH 43606 USA

**Keywords:** Dahomey basin, Bitumen, Seeps, Contamination, DNAPL, Hydrocarbon, Geogenic

## Abstract

The impact of bitumen components on soil and groundwater resources is of environmental importance. Contaminants’ influx into the environment from bitumen components through anthropogenic activities such as exploration, mining, transportation, and usage of bitumen in all its forms have been reported globally. However, gaps exist in the geogenic occurrence of bitumen in the shallow subsurface such as in southwest Nigeria, contaminating the soil and groundwater resources. This review presents in situ bitumen seeps as a source of geogenic soil and groundwater contaminants in southwestern Nigeria. We conducted a systematic review of literatures based on defined selection criteria. We derived information on the state of knowledge about bitumen seep occurrences and distribution in southwestern Nigeria. Also, the processes that exacerbate bitumen contaminants’ influx into soil and groundwater were enunciated. At the same time, case examples highlighted areas for possible in situ bitumen contamination studies in Nigeria. The results of this review showed that a multidisciplinary approach has been employed to assess and monitor the contaminants resulting from the various activities involving the exploitation and application of bitumen in Nigeria. These studies emphasize bitumen contaminants as emanating from anthropogenic sources. The results also suggested that bitumen studies have been mainly exploratory to improve the understanding of the economic potential of the hydrocarbon reserve. Also, recent advances in bitumen contaminants studies accounted for the heterogeneous nature of the bitumen. This allows for the optimized categorization of the mechanism and processes undergone by the different bitumen components when released as environmental contaminants. However, a knowledge gap exists in characterizing and understanding the effects of in situ bitumen seeps as a geogenic source of soil and groundwater contamination. This review identifies the possibility of geogenic soil and groundwater contamination by in situ bitumen seeps in the coastal plain sand of the Dahomey basin in southwestern Nigeria. The impact of the bitumen contaminants on the environment was discussed, while methods for accessing the occurrence and distribution of the bitumen contaminants were highlighted.

## Introduction

Bitumen is a dense mixture of heterogeneous hydrocarbon compounds produced from the temporal degradation of lighter crude oil (Brown et al., 2017; Speight, 2005; Stoyanovich et al., 2019). It is composed mainly of compounds denser than water under atmospheric  conditions known as dense non-aqueous liquids (DNAPLs). Although DNAPLs are insoluble in water, their solubility can be enhanced under favorable environmental conditions. These conditions include the energy of the environment, their residence time, the soil and aquifer hydraulic properties, and the presence of plant and organic matter (Redman et al., 1994). Unlike the lighter hydrocarbon compounds, DNAPLs like naphthalenes and polycyclic aromatic hydrocarbons (PAHs) are hard to detect in the environment since they can occur out of sight at the bottom of aquifers or water channels, from where they release toxins (Hollebone, 2015; Paliukaitė et al., 2014; Summons et al., 2007). Furthermore, the release of bitumen contaminants in the environment occurs through a series of physical, chemical, and sometimes biological processes (McKirdy et al., 2018; National Academies of Sciences and Engineering, 2016). These processes dictate the temporal and spatial distribution of DNAPLs contaminants. The presence of bitumen contaminants in soil and groundwater systems makes them unfit for farming and providing safe drinking water (Asubiojo & Adebiyi, 2014; Olajire et al., 2007; Rooney et al., 2012).

Globally, contaminants influx into the environment resulting from anthropogenic activities involving the mining, transportation, and industrial use of bitumen has necessitated the study of bitumen’s impact on soil and groundwater resources (Sun et al., 2017; Weinhold, 2011). Anthropogenic processes involving the exploration or exploitation of bitumen have resulted in different cases of environmental degradation. For example, phenol released from spilled bitumen or produce water used in the thermal recovery of bitumen from tar sand deposits contaminates groundwater in parts of Australia (Tang, 2003). In Alberta Canada, an increase in oil-sand mining, and in situ bitumen recovery has increased groundwater pollution risks in the oil sands region of Peace, Athabasca, and Beaver River basins (Timoney & Lee, 2009). In situ bitumen recovery processes such as steam-assisted gravity drainage (SAGD) has shown not only to increase the drawdown of available groundwater resources but also exacerbate the increase in groundwater salinity (Elsanabary et al., 2013; Miall, 2013). Anthropogenic operations involving the extraction and transportation of bitumen are responsible for releasing not less than 1400 compounds classified as contaminants (Weinhold, 2011). Some of these compounds, including sulfur oxides (SOX), nitrogen oxides (NOX), particulate matter, and toxic hydrocarbon compounds, pose significant challenges to the environment. Monitoring the environmental impacts of these compounds using reliable standards, e.g., the US EPA standards have been implemented with concentrations above regulated environmental thresholds reported from industrial areas associated with bitumen mining in Canada’s Alberta region (Dowdeswell et al., 2010; WBEA-HEMP, 2007). Also, bitumen diluents such as naphtha used in enhancing the transportation of viscous bitumen through pipelines comprised of low molecular weight aliphatic and aromatics (C3–C14) such as the BTEX and the naphthalenes (Sims et al., 1991). These diluents as well as other tailing products from the bitumen extraction process are accumulated in tailing ponds from whence leaching of hydrocarbon and heavy metal contaminants into soil and groundwater has been reported (Ahad et al., 2018; Fennell & Arciszewski, 2019; Roy et al., 2016).

Bitumen seeps formed, where liquid or semi-solid petroleum commonly infused with gaseous hydrocarbons escapes at a low rate to the Earth’s surface from shallow bitumen deposits (McKirdy et al., 2018), constitute a geogenic source of soil and groundwater contaminants. The escaping bitumen undergoes authigenic processes responsible for releasing at least 600,000 metric tons of oil into the global aquatic system (McKirdy et al., 2018). The interaction of natural bitumen seeps with groundwater within the McMurray geological formation in Alberta, Canada, has been established with the presence of monoaromatic acids serving as a diagnostic tool in discriminating the impact of natural bitumen seeps on groundwater from anthropogenic bitumen contaminant sources (Hewitt et al., 2020; Milestone et al., 2021). In Nigeria, the concentration of total and polynuclear aromatic hydrocarbons observed in groundwater samples from shallow wells within the Nigerian bitumen belt was found to be above thresholds for human consumption (Gbadebo, 2010). This contamination is possibly the result of bitumen seepages within this area (Akinmosin et al., 2011). Although bitumen occurrence and impact on the environment have been reported from various regions of deposition globally (Flego et al., 2013; Summons et al., 2007), the emphasis has been on the anthropogenic release of bitumen-related contaminants due to mining and industrial applications of the hydrocarbon resource. In Nigeria, studies on shallow bitumen occurrences are focused on exploration and economic potentials (Adeyemi et al., 2013; Bata et al., 2015) with few acknowledgments of its impact on the environment.

**Fig. 1:**
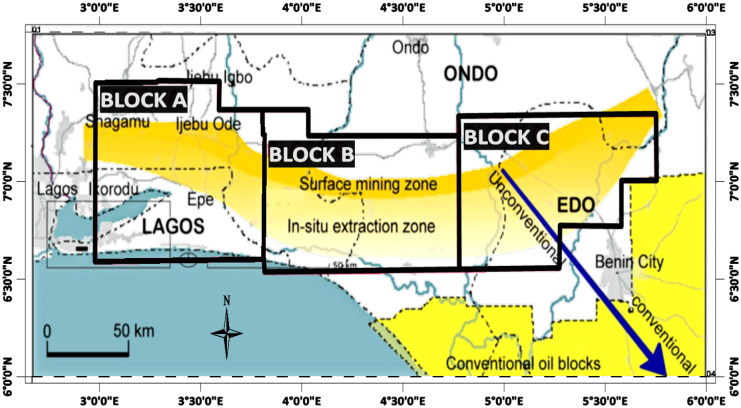
Map of the Nigerian bitumen belt showing Bitumen resources blocks (modified after Milos, 2015)

The assessment and management of bitumen contaminants’ impact on soil and groundwater have been the subject of various scientific research with emphasis on the contamination of groundwater resources as a result of bitumen’s exploration or mining activities (Asubiojo & Adebiyi, 2014). However, there is evidence showing bitumen seepages as a geogenic source of environmental contamination in the Dahomey Basin area of Southwestern Nigeria (Gbadebo, 2010). Previous authors agreed that bitumen seeps within the Nigerian sector of the Dahomey basin are sourced from two tar sand units known as X and Y horizons (Akinmosin & Imo, 2016; Falufosi & Osinowo, 2021). However, the mechanism of entry of bitumen contaminants into the soil and groundwater resources differs geographically. An up-dip migration of bitumen along the unconformity surfaces between the Dahomey basin sediments and the underlying basement rocks is widely reported to be responsible for the observed bitumen contaminations along the bitumen belt of the Nigerian sector of the Dahomey basin (Falufosi & Osinowo, 2021). However, a lack of adequate cap rocks for the bitumen source rock leading to oil migration is suspected to be responsible for the observed bitumen seep contaminant along the coastal region of the same basin flank (Akinmosin et al., 2011). Studies on bitumen contamination of soil and groundwater resources within the Nigerian sector of the Dahomey basin have emphasized anthropogenic activities as the cause of the contamination. These activities involving the exploration and exploitation of bitumen along the bitumen belt (Fig. 1) serve as the major source of the observed contaminations (Ogunsusi & Adeleke, 2019; Olajire et al., 2007), with only the component of the degraded oil present as evidence of contamination in the surface or near-surface environment (Akinmosin et al., 2019). More needs to be done, however, in understanding and mitigating geogenic bitumen contaminations as a result of in situ bitumen migration as seeps from compromised source rocks into soil and groundwater, particularly along the coastal area of the Nigerian sector of the Dahomey basin. This is important since most soluble and volatile components of this bitumen are preserved from degradation below the ground surface, where they percolate into soil and groundwater, thus creating a continuous source of soil and groundwater contamination. This review aims to present insitu bitumen seeps as a source of continuous geogenic soil and water contamination within the Nigerian sector of the Dahomey basin while identifying key processes acting upon the bitumen within the environment. These processes enhance the ease of bitumen contaminant influx into soil and groundwater resources. Also, methods applicable in understanding the dynamics and impacts of the geogenic bitumen contaminants on soil and groundwater resources were discussed with case examples.

## Study area

**Fig. 2:**
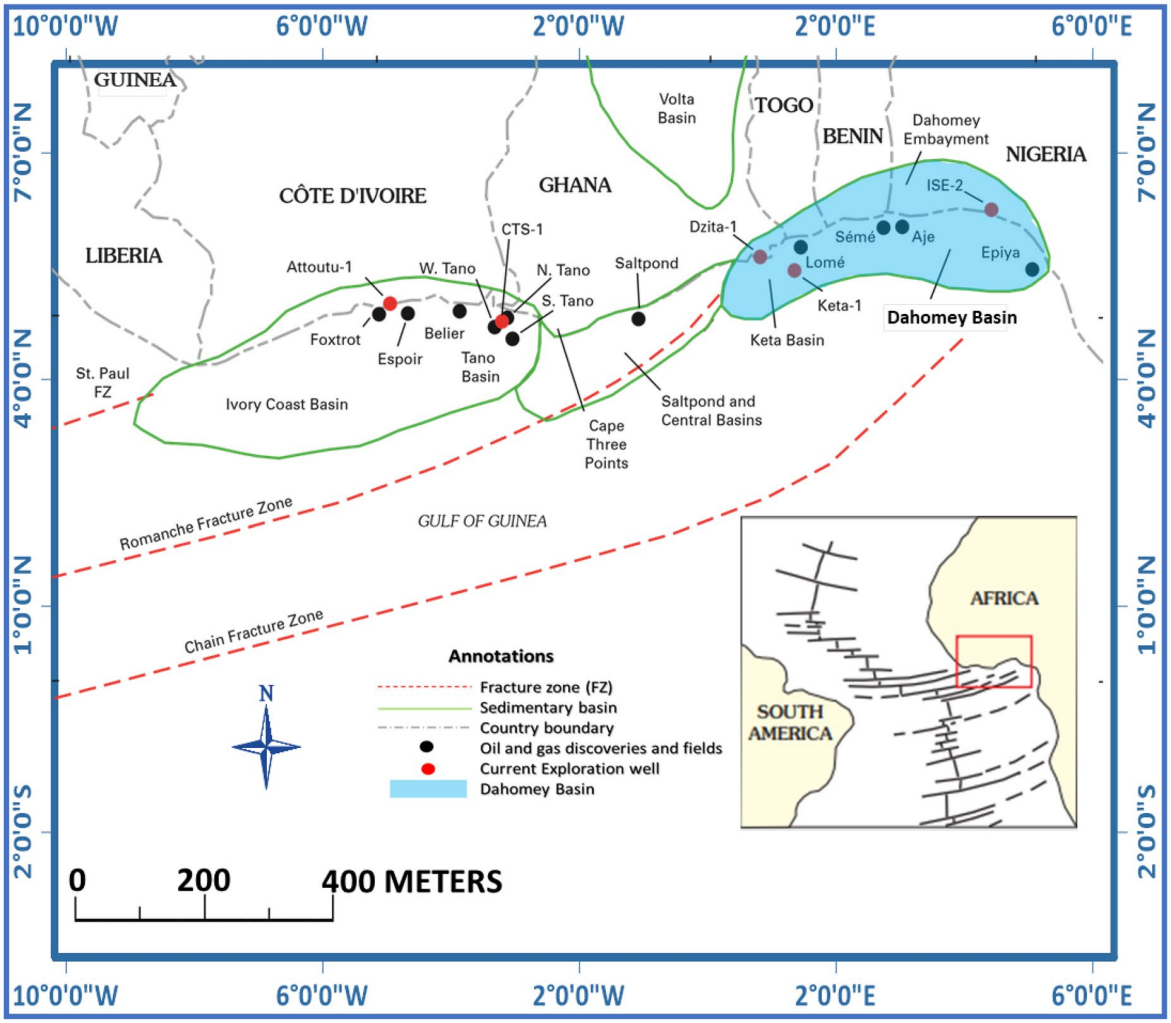
The Gulf of Guinea province of West Africa showing the Dahomey basin (modified after Brownfield & Charpentier, 2006; Kjemperud et al., 1992)

This study focuses on the Dahomey basin of southwestern Nigeria located within latitudes 6° 0′ 00″ to 7° 30′ 30″ and longitudes 2° 45′ 00″ to 5° 45′ 00″ (Fig. 2). The region is characterized by lowlands with few ridges’ characteristics of the tropical rain forest of southwestern Nigeria (Odunuga et al., 2013). Temperatures in the area are relatively high during the dry season, with highs of about 33 °C, while low temperatures are around 26 °C experienced during the rains, especially between July and August (Akintola, 1986). Rainfall distribution in the region varies from about 1000 mm in the western part to about 2000 mm in the eastern part, and the high rainfall promotes perennial tree growth with varying height (Oguntunde et al., 2011).

### Bitumen occurrence and distribution

Bitumen occurrences in Nigeria have been recorded from boreholes and seepages along river banks, cliff faces, slope breaks, road cuts, and farmlands (Milos, 2015; Omatshola & Adegoke, 1981). The bitumen which exists as one of the unconventional hydrocarbon resources within the Dahomey sedimentary basin outcrops in an east–west trend known as the bitumen belt of southwestern Nigeria (Milos, 2015). The transitioning from unconventional to conventional hydrocarbon resources within the sedimentary basin occur along an approximately north to south running gradient. The heaviest unconventional bitumen resources are generally found at the surface or near-surface environment in the northern section of the basin, while the lightest conventional crude oils are found in the deep subsurface towards the south (Fig. 1). This bitumen belt is about 5 to 8 km wide and stretches from Ijebu-Ode town in the west over a distance of about 120 km to the banks of the tributaries of Siluko River at Ofosu Village in the east, roughly spanning over four states, including Lagos, Ogun, Ondo, and Edo states of Nigeria (Enu, 1985; Falufosi & Osinowo, 2021; Milos, 2015). The regions where unconventional bitumen resources occur have largely remained under-explored, with little known about the precise location of the bitumen resources (Adegoke, 1980; Milos, 2015). Although, the bitumen belt has been divided into blocks categorizing the belt into zones based on probable mining methods (Fig. 1). Block B, with prominent evidence of bitumen surface outcrops, had been considered for in situ bitumen extraction through surface (open-pit) mining (Ako et al., 1983).

In southwestern Nigeria, bitumen is found within the Dahomey basin (Agagu, 1985), with the basin located in the Gulf of Guinea province (Fig. 2). The Dahomey basin extends along the Atlantic coast from southeast Ghana and terminates on Romanche Fracture Zone (RFZ) fault system. In southwestern Nigeria, the basin is separated from the Niger Delta basin in the east by the Benin Hinge Line. The Hinge line is the landward extension of the Atlantic chain fracture zone (Brownfield & Charpentier, 2006; Falufosi & Osinowo, 2021; Omatsola & Adegoke, 1981). The Dahomey basin is a marginal pull-apart basin whose development began in the Mesozoic (Jurassic–Cretaceous) and was associated with the separation of the African plate from the South American plate (Burke et al., 1971; Klemme et al., 1975). Subsidence in the underlying Precambrian Basement Complex rocks triggered sedimentation in the Early Cretaceous, with the sedimentation starting before the breakaway of the African continent from the South American continent, thus justifying the similarities in the sedimentary sequences observed along the marginal basins of West African coasts and most of the Brazilian basins (Omatsola & Adegoke, 1981).

**Fig. 3:**
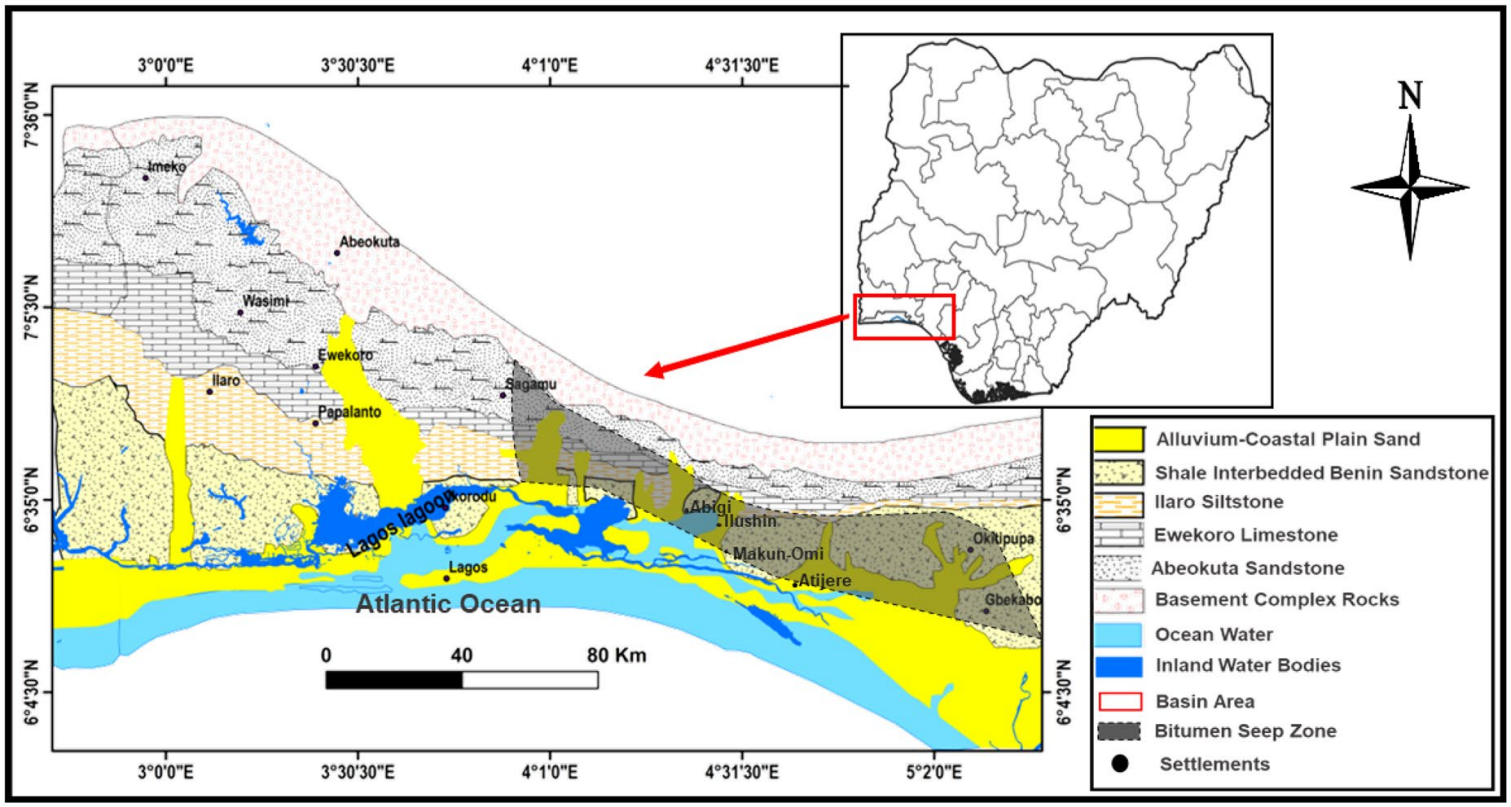
Geological map of Nigerian Sector of the Dahomey Basin showing zones of seeping bitumen outcrop along the Transition Margin from sedimentary to Basement Complex Rocks (modified after Agagu, 1985 and Enu, 1985)

**Fig. 4:**
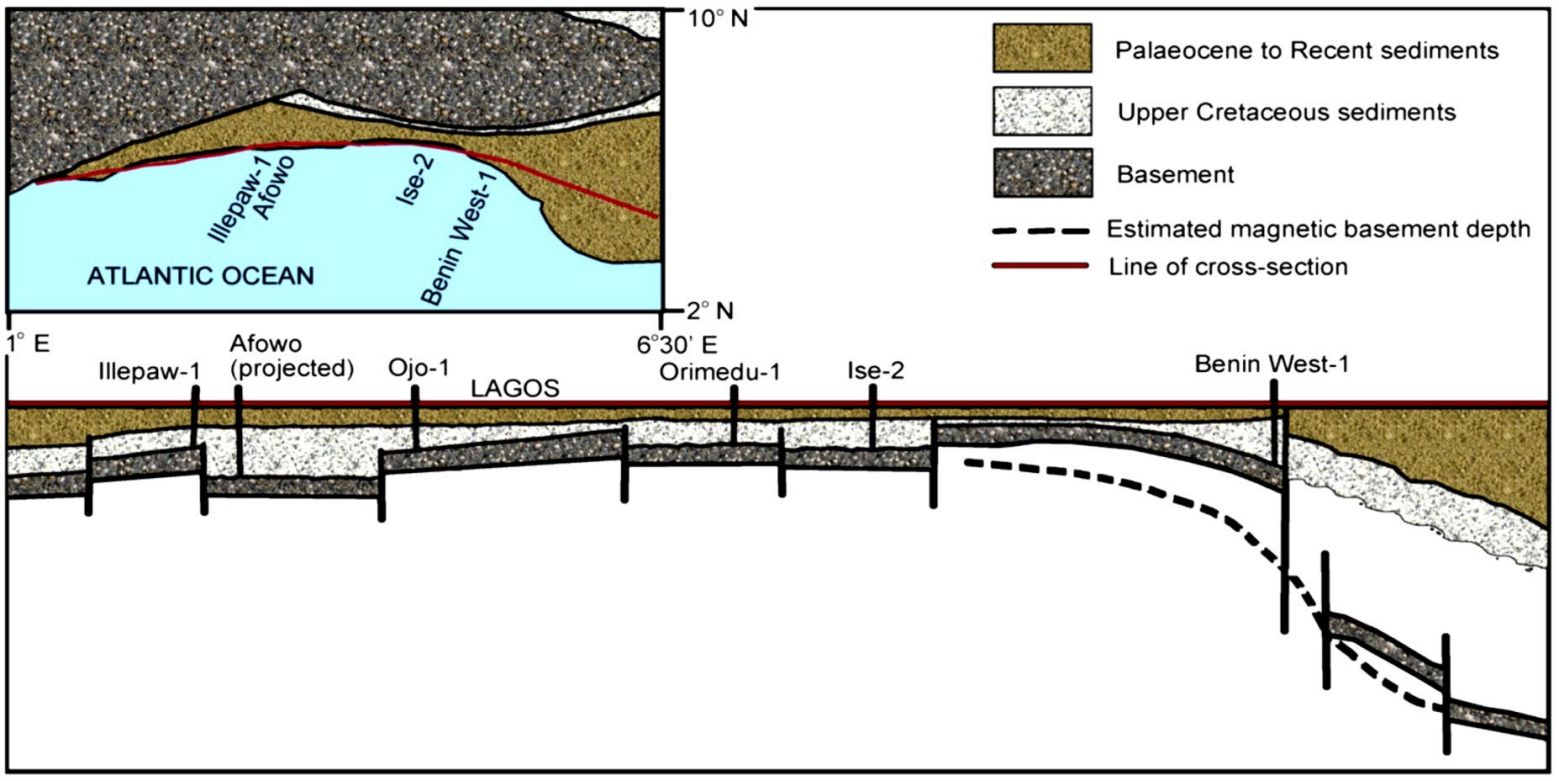
East–west cross-sectional line showing the series of horst and graben underlying the Dahomey basin (Falufosi & Osinowo, 2021)

The sedimentary sequence within the Dahomey basin is of Cretaceous to Recent in age as summarized in Table 1, and it unconformably overly the Precambrian Basement Complex rocks (Fig. 3), with information from well-logs showing that the basin is composed of a series of horsts and grabens (Fig. 4) which are series of normal faults serving as conduits for hydrocarbon migration (Falufosi & Osinowo, 2021). The contribution of these faults to the distribution of bitumen as seeps has been identified through geophysical investigations, with the use of electrical resistivity tomography. Akinmosin et al. (2011) observed that the presence of a normal fault with a throw of about 20 m can be credited for bitumen seepages around Ijebu Imeri area within the Nigerian sector of the Dahomey basin.

**Table 1:** Summary of the geology of Dahomey basin (modified after Agagu, 1985 and Enu, 1985)

Period	Age	Formation	Lithology
Quaternary	Recent	Alluvium	
Tertiary	Pleistocene-Oligocene	Coastal Plain Sand	
	Eocene	Ilaro	
		Oshoshun	
	Palaeocene	Akinbo	Shale
		Ewekoro	Limestone
Late-Cretaceous	Maastrichtian	Araromi	Shale/fine-sand/limestone
	Turonian	Afowo	Oil/tar-sand
	Neocomian	Ise	Sand (clean water aquifer)
Precambrian		Basement Complex Rocks

The oldest sediments within the Nigeria sector of the Dahomey basin are Late-Cretaceous in age consisting of grits, sandstones, and mudstones overlain by marginal to marine sands of the Maastrichtian age, here we have the Afowo Formation renowned for hosting tar-sand or bitumen deposits (Akinmosin et al., 2015; Ako et al., 1983; Okosun, 1990). The Cretaceous sediments are conformably overlain from west to east by Paleocene shale interbed with limestones and marl with the shale suggested to be a hydrocarbon source rock (Nton et al., 2003). The next assemblage overlying the Paleocene sediments are marine mudstones, shales interbedded with sand, and transitional to continental sands (Agagu, 1985; Enu, 1985; Falufosi & Osinowo, 2021; Jones & Hockey, 1964). Lastly, the sediments are overlain onshore with transitional to continental sands of Oligocene to Recent age (Billman, 1976; Fayose, 1970; Omatsola & Adegoke, 1981). The bitumen deposits found within the Maastrichtian Afowo sands are reported to be the source of observed bitumen seeps within the Nigerian sector of the Dahomey Basin (Enu, 1985). The lack of good cap-rock, as well as the shallow depth to the bitumen beds which ranges between 1 and 45 m along the transition zone at the northmost end of the basin (Enu, 1985), has been ascribed to result in up-dip migration of the bitumen units into near surface or surface environments where they are observed as seeps (Ekweozor & Nwacukwu, 1989). This up-dip migration northward is possible as the bitumen beds are dipping between 100 and 120 in a north–south direction (Enu, 1985). Also, the weathering of cap-rocks and other overlying units has exposed bitumen bearing beds along the transition zone to further surface migration and degradation (Akinmosin & Imo, 2016).

### Near-surface bitumen seeps

**Fig. 5:**
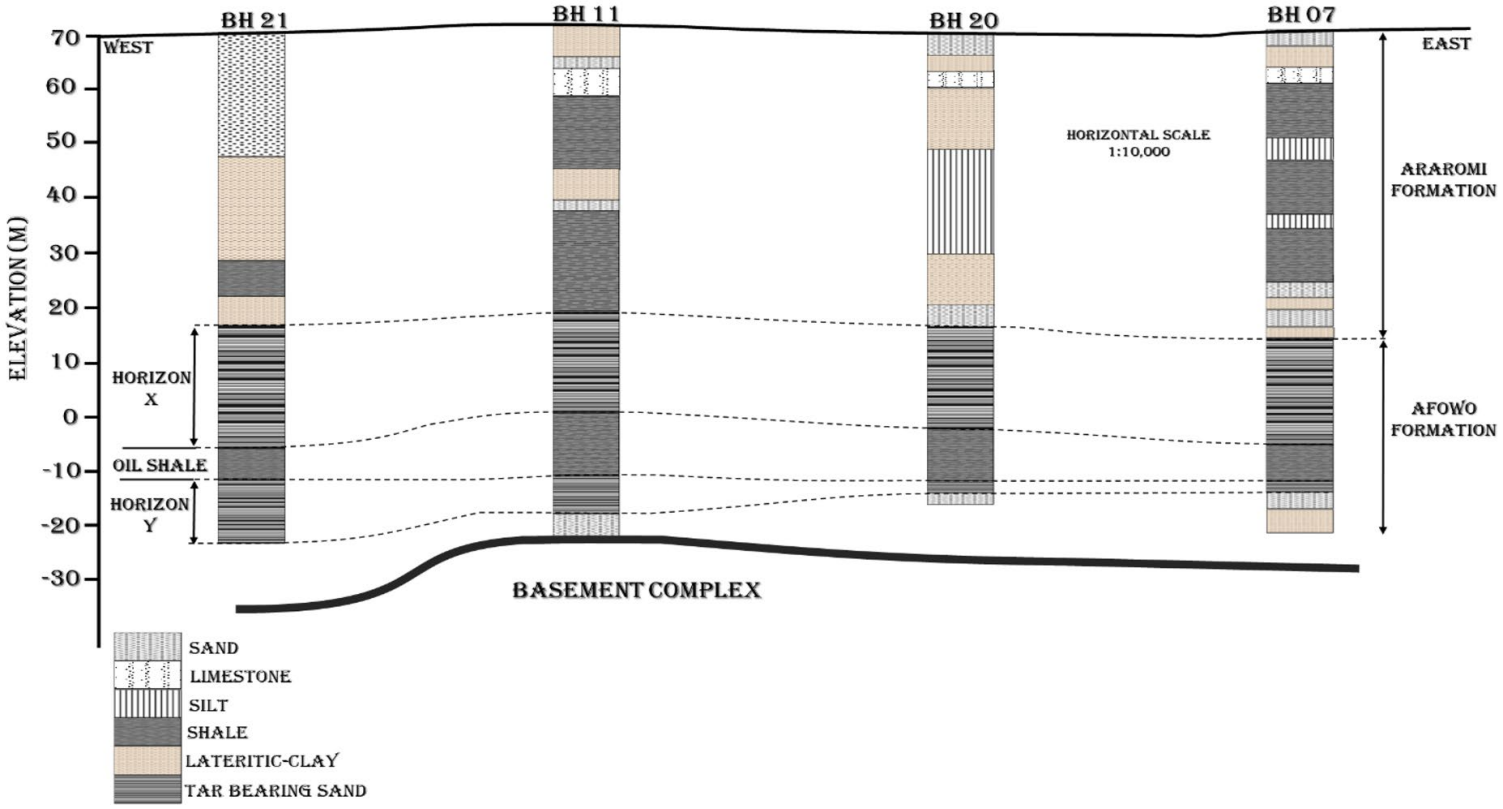
East–west geological cross section showing bitumen horizons of eastern Dahomey basin (modified after Akinmosin & Melifonwu, 2018; Ekweozor & Nwachukwu, 1989)

Bitumen seepages are suspected to be formed as a result of material transfer between shallow hydrocarbon reservoirs and the surface environment through conduits such as aquifers, shallow bedrock valleys, near-surface unconformities, faults, and fractures (Hein, 2006). In the Dahomey basin (Fig. 3), bitumen seeps are sourced from X and Y horizons within the Afowo Formation (Fig. 5), which are bitumen bearing sand units (Adegoke, 1980). In the northmost sections of the basin where the X horizon is completely eroded and the depth to the basement is relatively shallow, the prevailing hypothesis is that the oil seeps are sourced from an up-dip migration of oil from offshore dipping Y horizon (Enu, 1985). However, there are insufficient geological evidence to justify this hypothesis and there has been no laboratory or field scale experiments to access its plausibility. Recent researches ascribe the formation of bitumen seepages along the northmost part of the Nigerian sector of the Dahomey basin to normal faults present in the near surface region of the area acting as conduits for bitumen migration (Akinmosin et al., 2011). These normal faults are believed to be offshoots of the series of horst and graben that characterize the base of the basin (Brownfield & Charpentier, 2006). Bitumen within the southern coastal area of the basin is believed to be sourced from the two sedimentary horizons X and Y of the Afowo formation (Fig. 5) as observed from borehole records (Akinmosin & Melifonwu, 2018). The horizon X is suspected to be the major contributor to bitumen seepages within the area base on its proximity to the surface or near surface environment (Akinmosin & Imo, 2016; Emmanuel & Ajibade 2014; Ekweozor & Nwachukwu, 1989; Enu, 1985; Tomori et al., 2016) as well as the absence of a suitable caprock. The presence of the oil shale between horizons X and Y could serve as cap rock for bitumen in horizon Y. The integrity of the caprock would however be weaker in areas where it is less thick.

The bitumen seeps observed at the northmost part of the Nigerian Flank of the Dahomey basin are highly degraded due to exposure of the bitumen horizons to weathering (Ako & Enu, 1990; Ekweozor & Nwachukwu, 1989). Nigerian bitumen is described as a geological material susceptible to weathering at the surface or near-surface environment, with the process catalyzed by oxygen, flowing water, bacteria, and mining activities (Adeyemi et al., 2013). These bitumen degradation processes result in removing the lighter oil components leaving behind unreactive residual oil, which is immobile in the environment (Geng et al., 2014). Thus, the bitumen seeps in the northmost part of the Dahomey basin has been described as having minimal impact on soil and groundwater pollution due to high level of degradation (Asubiojo & Adebiyi, 2014; Gbadebo, 2010). Conversely, when the lighter constituents of seeping bitumen are present in the environment, they are introduced into the soil and groundwater resources by various environmental processes (Boufadel et al., 2010; Chapelle, 2001; King et al., 2014; Prince et al., 2003), resulting in the interaction of the bitumen components with soil and groundwater. This is possible when the seeping bitumen is not completely degraded and has its lighter components intact, as in the southern coastal region of the basin. Here, the adsorption and dissolution of the seeping bitumen components into soil and groundwater, respectively, can be seen in the surface water bodies, which are in sync with the aquifer system. Bitumen seeps within the southern coastal area of the Nigeria sector of the Dahomey basin are poorly reported in the literature, with evidence of bitumen occurrence mostly restricted to borehole core samples retrieved by the defunct Nigerian Bitumen Corporation (Agagu, 1985; Ako & Enu, 1990). As the occurrence of bitumen seepages within the coastal region of the Dahomey basin is poorly discussed in the literature, little is known about the mode of occurrence and distribution of the bitumen seeps. Hence, the mechanism of interaction of the bitumen seeps with soil and groundwater resources within this region is scientifically unproven.

## Review methods

### Review procedure and data sources

**Fig. 6:**
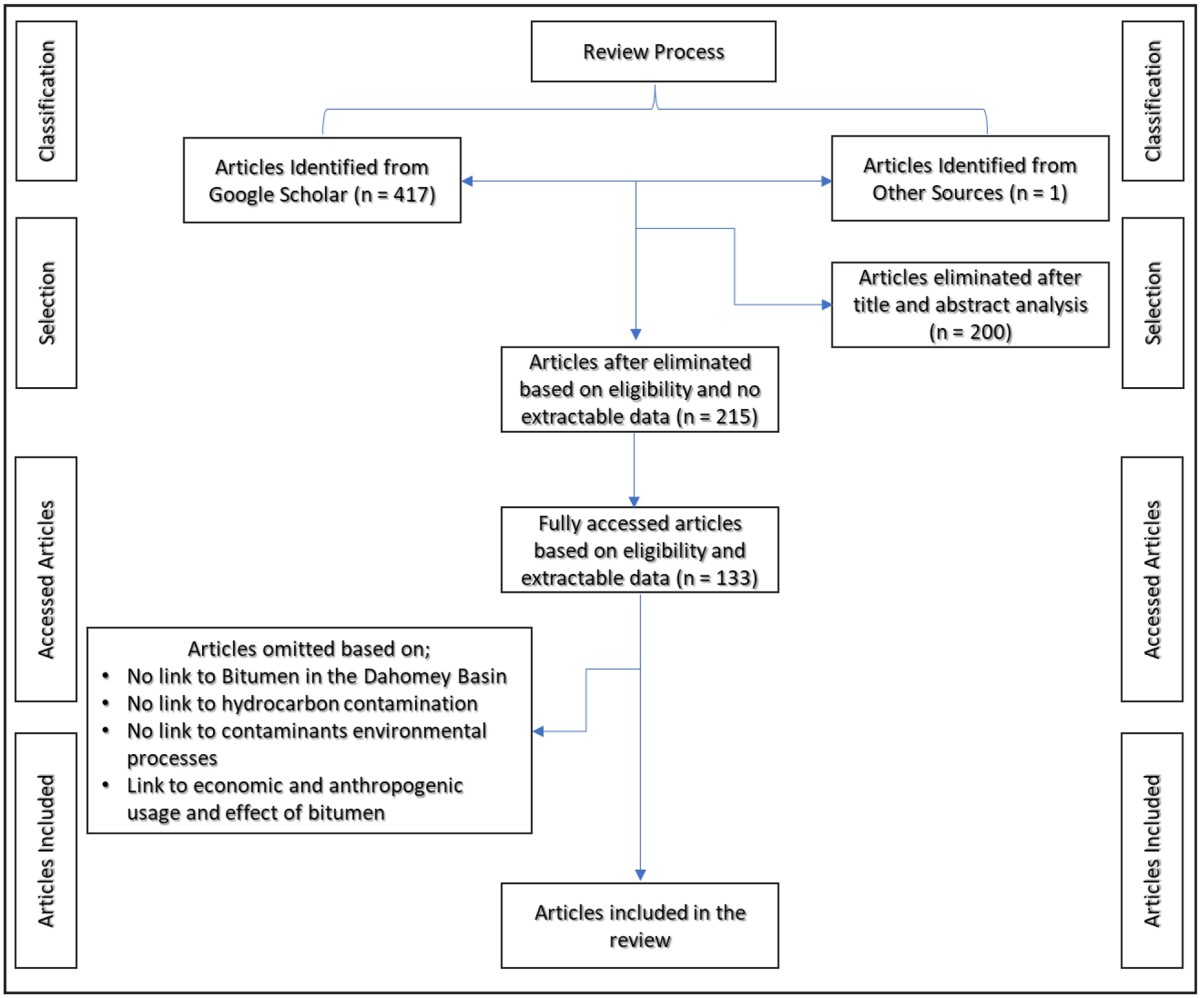
A schematic of the review methodology employed for this article

The method used in this review complied with the Preferred Reporting Items for Systematic reviews and Meta-Analyses (PRISMA) statement 2009 (Page et al., 2021). The review was written after consulting open access literature written about the bitumen deposits within the Dahomey basin of southwestern Nigeria. Database article querying was guided using definite keywords (Wali & Alias, 2020). The keywords serve as search terms to access articles discussing the origin, composition, and mode of occurrence of the bitumen hydrocarbon resources from 1964 to 2021 (Table 2). A total of 417 articles of potential relevance to the review title were accessed through Google Scholar (Fig. 6). An exclusion approach was used to select regional references (Wali et al., 2020). The lack of sufficient research articles on the environmental impact of in situ bitumen resources within the Dahomey basin of southwestern Nigeria necessitated considering articles from other global bitumen deposits to provide succinct information where the regional articles were lacking.

**Table 2:** Search terms used in querying the database and in article classification (After Wali & Alias, 2020)

Term classification	Search terms
In situ bitumen contamination	Hydrocarbon contamination, bitumen DNAPL, bitumen environmental processes, fate of environmental contaminants
Bitumen in the Dahomey basin	Bitumen resources, Dahomey basin, bitumen seepages, bitumen composition (1964 to 2022)

The content of the review is grouped into sections as follows:

(A) Geology of the study area
  This describes the state of knowledge on the history and mode of occurrence of bitumen deposits and seepages within the basin of interest. Stratigraphic and structural controls on bitumen seep formation were discussed.
(B) Environmental importance of seeping bitumen
  Here, the possibility of bitumen components acting as a source of dense non-aqueous phase liquid (DNAPL) contaminants in the environment is discussed. This was described from the chemical composition of seeping bitumen from the study area with references to areas where bitumen contaminations have been reported globally.
(C) Bitumen contamination processes
  Bitumen within the surface or near-surface environment has been proven to be a source of soil and groundwater contaminant. This is made possible by various processes undergone by the bitumen components in the environment. This section describes the types of processes resulting in the contamination of soil and groundwater in the environment and the corresponding results of such contamination from in situ bitumen contamination observed within the study area and with references to similar scenarios in other regions with known bitumen contaminations.
(D) Case history
  This aspect highlights and discusses key findings from relevant literature on the origin, occurrence, and composition of bitumen seeps within the study area, while also pointing to several works done so far in understanding the environmental impact of the seeping bitumen components on soil and groundwater resources.
(E) Conclusions
  The review concluded by identifying the different methods employed within the study area to assess the effect of bitumen-sourced contaminants on soil and groundwater resources while drawing a comparison with work done globally. The article identifies knowledge gaps in previous studies on bitumen contamination within the southwestern regions of Nigeria. This review, therefore, presents a unique opportunity for further studies.

## Results and discussions

### Bitumen as a source of DNAPL contamination

**Fig. 7:**
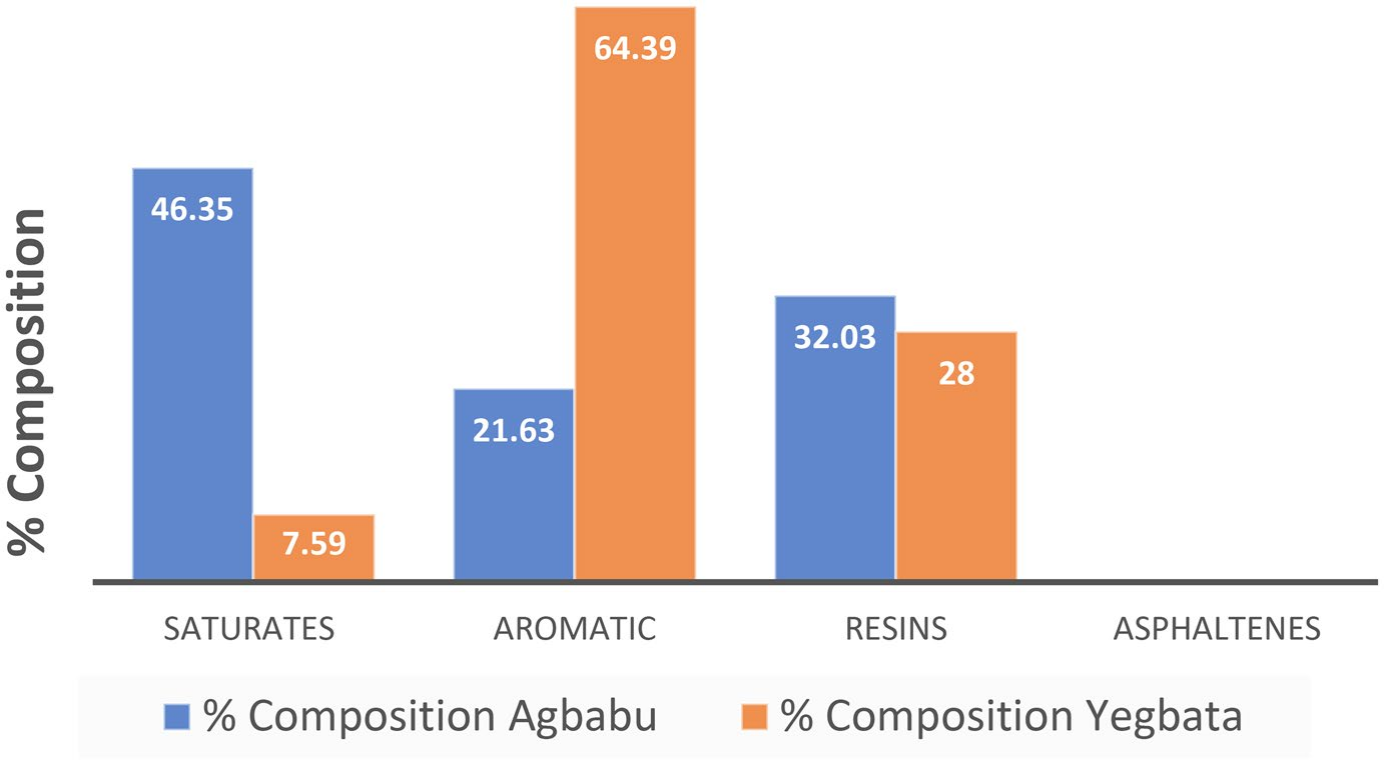
Percentage component composition of Agbabu and Yegbata bitumen samples from the Dahomey basin (modified from Ogiriki et al., 2018)

Bitumen typically consists mainly of hydrocarbons categorized as saturates, aromatics, resins, and asphaltenes (Paliukaitė et al., 2014; Remišová & Holý, 2018; Stoyanovich et al., 2019). Analysis of two samples of bitumen from different sites, namely Agbabu and Yegbata (Fig. 7), within the eastern flank of the Dahomey basin in southwestern Nigeria reported percentage component composition as 46.35% and 7.59% saturates, 21.63% and 64.39% aromatics, and 32.03% and 28.01% resins (Ogiriki et al., 2018).

These components exhibit variability in physiochemical properties, which in turn affect their ease of becoming soil and groundwater contaminants within the environment. Globally, the interaction of the various components of bitumen as contaminants in the environment has been reported (Ahad et al., 2013, 2020; Fennell & Arciszewski, 2019; Stasik et al., 2015; Sun et al., 2017). Amongst the listed components of bitumen, aromatic hydrocarbons are of greater environmental importance as contaminants (Klungsoyr, 1999; Scott et al., 2012). This is because polycyclic aromatic hydrocarbons (PAHs) are known for their toxicity and persistence in the environment, as they can result in mutagenicity and carcinogenicity (Chen et al., 2004; Woo et al., 2004). The United States Environmental Protection Agency (US EPA) and the European Union (EU) have identified 16 PAHs in their priority pollutant list. These priority PAHs have low aqueous solubilities (0.003–0.34 mg/L), high octanol–water partition coefficient (log Kow = 3.4–7.6), and high organic carbon partition coefficient (Log Koc Organic carbon 3.86–6.74). They exhibit a low dissolution rate in water, and there is a higher rate of accumulation in sediments (Eszter et al., 2004; Hans Peter et al., 2009). There is also the possibility of re-introducing these PAHs into the environment from contaminated sediments (Conrad et al., 1992; Mayer & Miller, 1993). Within the Dahomey basin in southwestern Nigeria, where bitumen seeps into soil and water resources in the environment, the presence of anomalous levels of bitumen-sourced polycyclic aromatic hydrocarbons (PAHs) has been reported in soil and water samples (Fagbote & Olanipekun, 2010; Olajire et al., 2007; Ololade et al., 2012).

Unlike aromatic hydrocarbons or resins and asphaltenes, saturates are easily biodegradable. As such, easily metabolizable saturates are not found in the environment. While chemical analyses of samples from saturated hydrocarbon in deep-seated bitumen and crude oil show dominantly straight-chained saturated hydrocarbon, which is readily metabolizable, saturates from seeping bitumen in the environment are composed mainly of branched and cyclic hydrocarbons that are more resistant to biodegradation (Swarthout et al., 2015). This observed structural change occurs as a result of the reworking and degradation of the straight-chained hydrocarbon with proximity to the surface environment. Saturates found in the environment as contaminants are also described as aliphatic hydrocarbons and are reported in sediments and water samples within the bitumen belt of the Dahomey basin (Adedosu et al., 2021; Fagbote, 2013). Resins and asphaltenes are heavy and adhesive components of bitumen, having between 30 to 70 carbon atoms and a wide range of structures, with their molecules tending to cluster into larger multimolecular aggregates (McKenna et al., 2013). They are completely insoluble in water, and as such, their presence in the environment poses no direct threat to groundwater resources. However, they cause ecotoxicity due to their effect on the floras and fauna components of the environment as a result of the formation of tar crust and resulting water repellency in sediments upon which they are released, thus creating a less than ideal environment for plant growth by reducing the rate of wetting and retention of water in soils (Doerr et al., 2000, Roy et al., 2003). Also, heteroatom such as nitrogen, sulfur, oxygen, and heavy metals commonly associated with the resin fraction of the bitumen have been reported in high relative abundances in bitumen-contaminated soil and water samples retrieved within the Dahomey basin (Atojunere, 2021; Ayandiran et al., 2018; Korosi et al., 2016).

### Bitumen contamination processes

Bitumen in the environment undergoes various chemical and physical property modifications with eventual loss and addition of new components through the process of weathering or degradation (Stasik et al., 2015; Stoyanovich et al., 2019). The environmental behavior of bitumen as regards degradation or weathering can be categorized into three based on the nature of the chemical and physical processes acting on the oil (National Academies of Sciences and Engineering, 2016).

#### Chemical processes

These result in the decomposition or alteration of the bitumen molecules, creating strains more viable as contaminants in the environment. They are processes that take place within a short to long period with varying products under different environmental conditions. The chemical processes of great importance undergone by bitumen in the environment leading to the breaking down or modification of the bitumen at a molecular level include photochemical oxidation and biodegradation. Photochemical oxidation leads to the breakdown or modification of bitumen components into strains more susceptible to environmental modifications, such as the production of bitumen components with higher solubility in groundwater (D’Auria et al., 2009). Also, biodegradation leads to the removal of the lighter components of bitumen which are in turn washed into soil and groundwater as contaminants (Chapelle, 2001; King et al., 2014).

##### Photochemical oxidation

In the presence of sunlight and oxygen, bitumen components in the environment are oxidized to produce carbon dioxide, CO_2_ (greenhouse gas), and other oxygenated hydrocarbon molecules. D’Auria et al., (2009) show the susceptibility of bitumen components to photo-oxidation in a freshwater environment to be in the order aromatics > linear alkanes > branched alkanes, suggesting the removal of the lighter aromatic components and the formation of relatively abundant residual resin and asphaltene molecules (Prince et al., 2003). Photochemical oxidation of bitumen in the environment can be observed through the presence of carboxylic acids and alcohols in ground and surface water as they are soluble oxygenated compounds released as by-products leaving behind heavy bitumen compounds which persist in the environment (Chapelle, 2001; Aeppli et al., 2012). Also, photosensitive PAHs have been known to undergo photochemical enhancement in sunlight which in turn increases their toxicity in the environment (Barron et al., 2003). The photooxidation products of bitumen have been observed in soil and sediment samples analyzed for suspected bitumen contamination within the Dahomey basin, with the presence of carboxylic acids and alcohols resulting from the photochemical oxidation of bitumen suspected for inhibiting biodegradation activities of bacteria (Olabemiwo et al., 2011).

##### Biodegradation

Biodegradation occurs through the metabolic activities of certain bacteria on bitumen components in the environment in the presence or absence of oxygen, with the latter being prominent (Boufadel et al., 2010). In situ seeping bitumen is classed as degraded oil, with most of its volatile and lighter components lost in transit, therefore, the presence of abundant heavy molecular hydrocarbon in bitumen slows down the rate of biodegradation. US Environmental Protection Agency (USEPA) experimented with the biodegradation of bitumen in sediments under favorable conditions for bacterial activities, and the result shows a significant drop in the rate of biodegradation with a drop in the concentration of lighter hydrocarbon components (USEPA, 2013), thus suggesting the negligible effect of biodegradation on the heavy oil components of bitumen in the environment. Evidence from studies of biodegradation of Agbabu bitumen within the Dahomey basin and bitumen-polluted water from tailing ponds within the Canadian Athabasca oil sands mining operations show that biodegradation removes the lighter and more volatile components of bitumen leaving behind residues that persist in the environment (Ahad et al., 2018; Olabemiwo et al., 2011).

#### Physicochemical processes

Physicochemical partitioning processes include evaporation and dissolution of bitumen components in the environment. These processes occur mainly with the soluble or volatile phases of the seeping bitumen and do not result in changes to the molecular structures of the bitumen components (National Academies of Sciences and Engineering, 2016).

##### Evaporation

The loss of volatile compounds in bitumen due to evaporation when the oil comes in contact with the atmosphere leads to the formation of dense residual bitumen compounds which sinks to the bottom of water bodies in the environment. Evaporation of volatile bitumen compounds is controlled by; concentrations of the volatile compounds in the oil, ambient environmental conditions including exposed surface area and volume, the temperature of the oil, water, and air; and velocities of the wind current (Hamoda et al., 1989). Evaporative loss of lighter bitumen components will result in increased density and submergence of residual non-volatile components and an increase in the adhesiveness of the persistent hydrocarbon components to the soil/sediments in the environment (Guma et al., 2012; King et al., 2014). Although evidence of evaporative losses of bitumen components is sparsely discussed in research carried out on bitumen contaminations within the Dahomey basin, the occurrence of this process can be deduced from the presence of degraded bitumen observed within the basin’s bitumen outcrop belt (Agagu, 1985; Enu, 1985; Eruteya et al., 2021).

##### Dissolution

Although bitumen as a DNAPL source is insoluble in water, it, however, consists of components with variable solubility in water (Lapidus et al., 2018). When bitumen seeping into the surface environment comes in contact with ground or surface water, components that are more soluble in water will be lost by dissolution (Rivett & Feenstra, 2005). Although most soluble components are volatile and are also prone to be lost by evaporation, bitumen seeps are, however, propagated in the subsurface with little or no contact with the atmosphere for an extended time, such as in the Deepwater Horizon oil spill (Reddy et al., 2012). Volatile components of bitumen seeps undergo dissolution in ground and surface water more readily than evaporation loss, and as such, the study of the fate and transportation of dissolve bitumen seeps components are of paramount importance when considering the impact of in situ bitumen seeps on soil and groundwater resources. For example, bitumen-sourced compounds such as the polycyclic aromatic hydrocarbon were observed through the elemental analysis of water samples from surface and groundwater reserves within the Nigerian flank of the Dahomey basin (Asubiojo & Adebiyi, 2011; Ayandiran et al., 2018; Atojunere & Ogedengbe, 2019; Fagbote et al., 2014). Also, the presence of polynuclear aromatic hydrocarbons in water samples analyzed for the impact of bitumen contaminants within the Ogun block aspect of the Nigerian bitumen belt indicates the dissolution of the bitumen sourced DNAPLs within the water resources of interest in the Dahomey basin (Gbadebo, 2010).

#### Physical processes

**Fig. 8:**
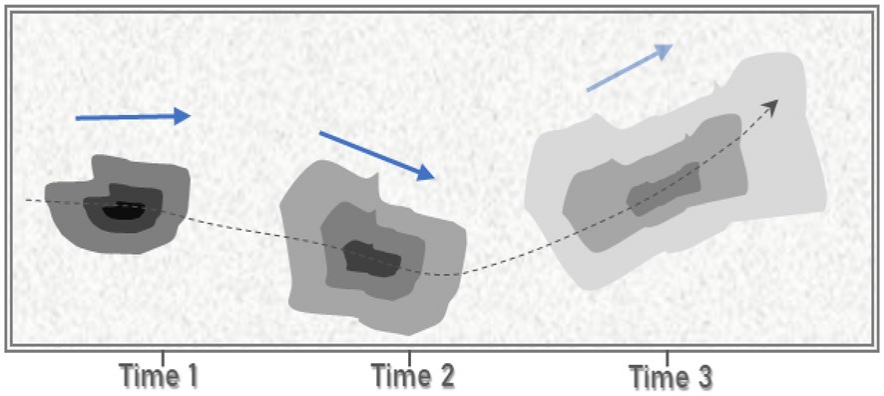
Schematic two-dimensional section through a contaminant plume moving and spreading with time. Blue arrows show the velocity path at each time, and the dashed line shows the path taken by the contaminant mass. The concentration levels are denoted by the degree of shading, with darker shades representing higher concentrations. The contaminant always disperses in the direction of flow. A varying flow direction causes the plume to grow wider in addition to lengthening (modified after Fitts, 2013)

These processes do not affect the molecular composition of seeping bitumen but are integral mechanisms in the fate and transport of bitumen components in the environment. They include spreading, emulsification, adhesion, and sedimentation. All of these changes the physical properties and behavior of the oil but do not always partition it between phases or change its molecular structure (National Academies of Sciences and Engineering, 2016). These physical processes are responsible for the redistribution or transportation of contaminants such as bitumen components in the environments (Fitts, 2013; Giadom et al., 2015), whereas they do not result in change in the composition of the contaminants, the concentration of the contaminant can be varied over time (Fig. 8).

##### Spreading

This occurs both on land and on water, but mostly on the water when high water surface tension prevents the submergence of oil from bitumen or other crude oil sources. The oil on the surface of the water forms a sheen which is a visible thin layer of oil immiscible with water spreading along the flow path of the water (Fay, 1971; Hoult, 1972). These sheens are from submerged residual bitumen components and bitumen seeps below the water table. Generally, the physical process of bitumen contaminant spreading is expected to be controlled by the mechanism of solute transport in the environment, such as advection, diffusion and mechanical mixing (dispersion), and sorption (Gerba et al., 2015).

**Fig. 9:**
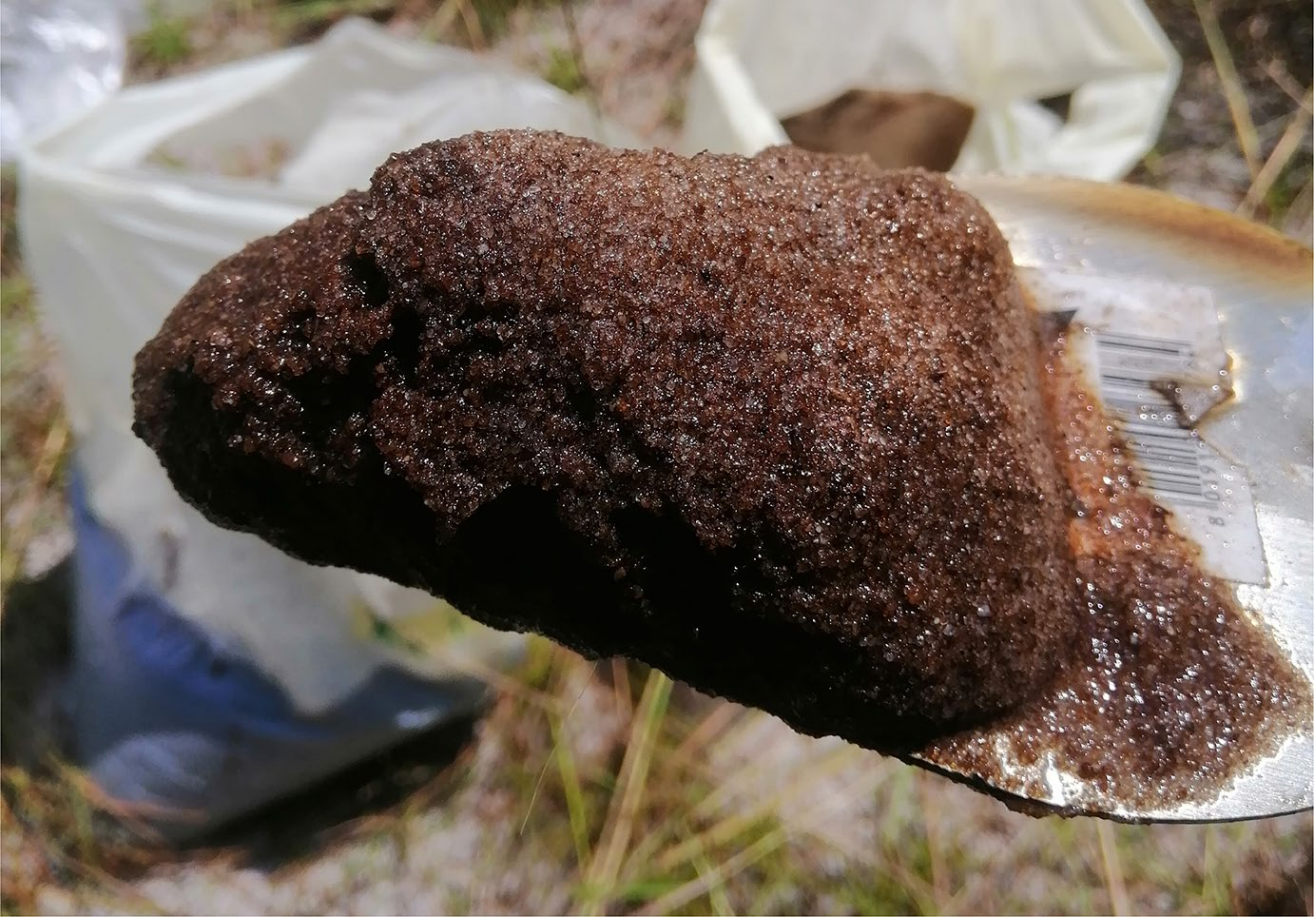
Sandy aquifer material showing evidence of sorbed bitumen contaminant within the coastal community of Nigerian sector of the Dahomey basin

1. Advection
  After the dissolution of bitumen components in moving water, the emanating contaminants are generally transported in the direction of flow due to the movement of mass entrained in the flow (Gerba et al., 2015). The flux of the dissolved contaminant due to advective spreading can be determined using the expression:
  1 $${\mathrm F}_{\mathrm{ax}}={\mathrm q}_{\mathrm x}\mathrm C$$
  where Fax is the advective flux of contaminant mass in the longitudinal flow direction, q_x_ is specific discharge in the longitudinal flow direction, and C is the contaminant’s concentration (Fitts, 2013). This expression defines the advective flux under steady-state laminar flow conditions. For hydrocarbon contaminants in water, the rate of advection depends on the velocity of flow of the transporting groundwater (Giadom et al., 2015). While research on the advective transportation of bitumen contaminants in groundwater within the Nigerian sector of the Dahomey basin is poorly reported, the transportation of hydrocarbon contaminants through advection in the neighboring Niger-Delta basin has been studied. Giadom et al. (2015) determined groundwater flow velocity in a hydrocarbon contaminated sandy aquifer through a tracer test to be 121 m/day, suggesting relative ease of contaminant migration through advection although attenuation of the contaminant transport by the presence of clay in the aquifer is expected. Similarly, modeling tracer migration through advection was suggested as an index for determining the advective flux of soluble hydrocarbon contaminant in a fine sand aquifer located within the Niger-Delta basin (Ugbena et al., 2020) thus providing a template for such studies within the Dahomey basin.
2. Dispersion
  The entrainment of bitumen oil droplets in a water body, a process driven by the interfacial tension between hydrocarbon and water, bitumen component viscosity, and the mixing energy driven by wind, currents, or tides results in hydrocarbon contaminant dispersion (National Research Council, 2005). This dispersion connotes the spreading of bitumen components about the center of the oil droplet as a result of molecular diffusion and nonuniform flow fields (Brusseau, 2019), As oil droplets are moved through advection, the sizes of the oil droplets increase through dispersion. The distribution of bitumen components dispersed in water is controlled by their droplet sizes. Larger droplets are more buoyant than smaller droplets and, as such, float to the surface whether they were released underwater or released at the surface, as observed in the Deepwater Horizon spill in the Gulf of Mexico, where large crude oil droplets (> 1.0 mm) released at depth, rose almost vertically and reached the surface within hours (Ryerson et al., 2012). Small bitumen oil droplets increase the risk of environmental contamination by seeping bitumen as a large proportion of small submerged droplets increase the chance of hydrocarbon dissolution in the water column, thus enhancing further redistribution of various possible hydrocarbon contaminants in surface or groundwater (Rahbeh & Mohtar, 2007; Reddy et al., 2012). Also, temporal and spatial variation in advective flow velocities will cause the contaminant plume to disperse in the direction of flow as contaminant components travel along the flow path at a different rate (Fig. 8). Spatial variation in flow velocities results in longitudinal dispersion, while traverse dispersion is a product of temporal changes in flow velocities in the general flow direction.
  Whereas dispersion as a result of molecular diffusion occurs when individual contaminant molecules spread from zones of higher concentrations to areas with lower concentrations, dispersion as a result of nonuniform flow occurs due to spatial and temporal variation in flow velocities. The latter is the major cause of contaminant plume dispersion. The rate of contaminant dispersion through mass diffusion termed diffusive mass flux can be expressed through Fick’s law in any direction of flow “x” as:
  2 $${\mathrm F}_{\mathrm{dx}}=-{\mathrm{nT}}_{\mathrm x}\mathrm D\frac{\partial c}{\partial x}$$
  where F_dx_ is the diffusive mass flux of the dissolved contaminant in the x direction, n is the porosity of the aquifer, T_x_ is the tortuosity of the contaminated water in the x direction, D is the molecular diffusion coefficient, and C is the contaminant’s concentration. Similar expressions would apply in the y and z directions. The flux of soluble hydrocarbon contaminant in any flow direction will be proportional to the concentration gradient in the same direction. The negative sign in Eq. (2) indicates the contaminant’s migration towards decreasing concentrations in the opposite direction to the concentration gradient (Fitts, 2013).
3. Sorption
  Sorption also influences the spread or transport of bitumen contaminants in the environment. The process can be adsorption or retention, which is the adhesion of bitumen molecules with the solid matrix of soil or groundwater aquifer serving as the transporting medium (Brusseau, 2019). Sorption critically results in the retardation of contaminant transportation by advection and dispersion mechanisms. So far, contaminated soil and aquifer grains are immobile while sorbed contaminant molecules remain immobile without being transported. In a scenario where contaminant sorption does not exist, the rate of advective–dispersive contaminant transport can be derived from the average linear flow velocity ‘v’. However, if the contaminant is sorb significantly, the rate of transportation is lower than”v” (Fitts, 2013). The sorption mechanism acting on the observed bitumen contaminant within the Dahomey basin is yet to be the subject of adequate scientific research, although the adsorption of bitumen contaminant into aquifer material has been observed in undocumented reports from borehole lithologic logs within the coastal area of the Dahomey basin in southwestern Nigeria (Fig. 9).

##### Emulsification

**Fig. 10:**
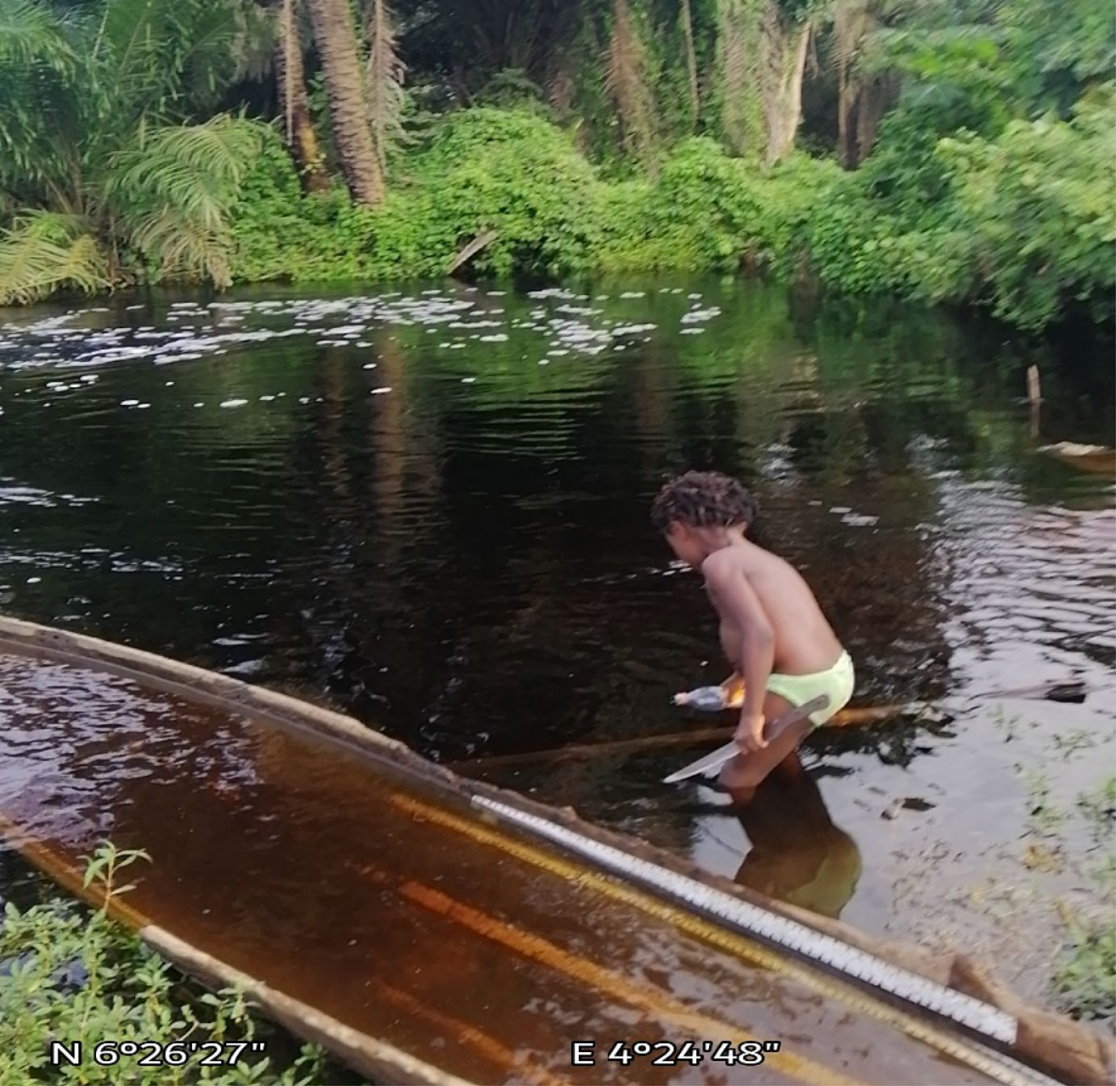
Reddish-brown coloration of surface water suspected to result from the emulsification of bitumen components in water at a coastal community within the eastern flank of the Dahomey basin

**Fig. 11:**
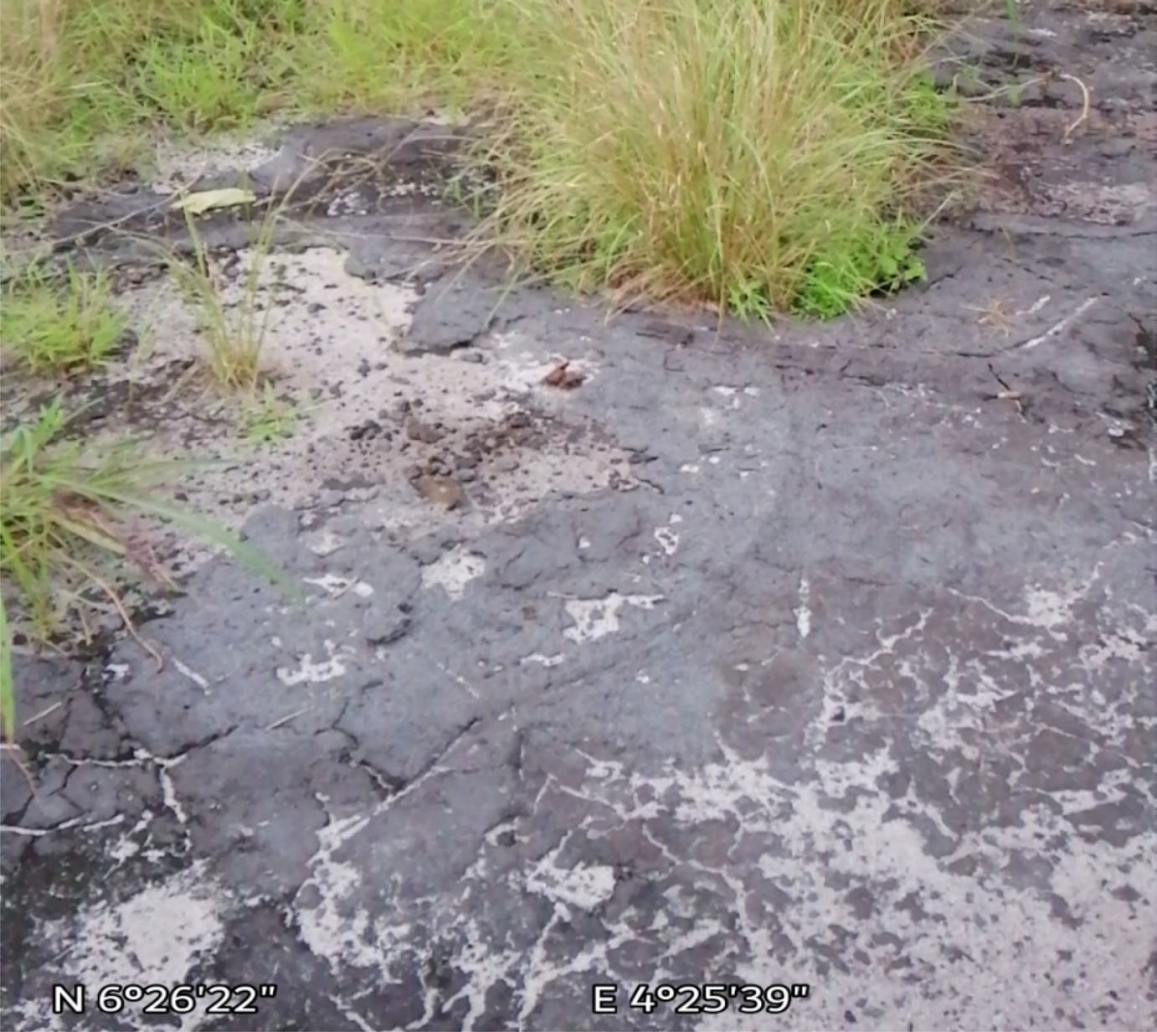
Product of stable emulsification of bitumen components (tar crust) at a coastal community within the eastern flank of the Dahomey basin

Also, seeping bitumen with relatively high viscosity compared to non-degraded oil undergoes mesostable or stable emulsification in the environment. Mesostable emulsions would be formed when small droplets of water are stabilized by a combination of the viscosity of bitumen and the interfacial action of asphaltenes and resins leading to increased viscosity of the original bitumen (Fingas & Fieldhouse, 2009). Mesostable emulsions are reddish-brown mixtures that generally break down within a few days into oil and water or emulsion remnants, while stable emulsions are also reddish-brown mixtures that appear to be nearly solid due to their high viscosity. They do not spread and could be found as mats on soils. Both mesostable and stable emulsification of bitumen has been observed within the Dahomey basin, with the reddish-brown appearance of surface water (Fig. 10) around communities along the coastal regions of southwestern Nigeria suspected to be due to the formation of mesostable emulsions by bitumen in the environment. Also, the formation of stable emulsion carpets known as tar crusts (Fig. 11) can be seen within the same vicinity. The formation of emulsions is significant where oil seeps into soil and water resource as emulsification substantially increases the actual volume of the seeping contaminant due to the addition of a significant amount of water. More importantly, oil in stable emulsions is difficult or impossible to disperse or burn (National Academies of Sciences and Engineering, 2016). Though mesostable emulsions are relatively easy to disperse, stable emulsions may take months or years to break down naturally. Emulsification also slows the processes of evaporation and biodegradation as well as the dissolution of soluble components of seeping bitumen.

##### Adhesion and sedimentation

Seeping bitumen in contact with the atmosphere suffers an evaporative loss of its volatile components, causing the residual heavy components to adhere strongly to soil, trees, plant, biota, and other organic matter in the environment (Fitzpatrick et al., 2015). The adhesion of residual bitumen components to sediments in the environment also leads to the formation of tar crusts (Fig. 11). Also worthy of note in the physical processes associated with seeping bitumen in the environment is the sedimentation of bitumen in water leading to the formation of oil-particle aggregates (OPAs) through the aggregation of oil with natural particulate matter. The formation of OPAs results in the submergence of an initially floating oil (Fitzpatrick et al., 2015). The presence of OPAs increases the concentration of submerged bitumen components, which can be a source of recurring bitumen contamination in surface or groundwater, and as such, they must be considered when assessing the impact of in-situ bitumen seeps on soil and groundwater resources in the environment.

### Case history of bitumen seeps in the Nigerian sector of the Dahomey basin

#### Origin

Understanding the source of observed bitumen seeps in the environment is important in developing a focused approach to their assessment and probable remediation in regions where such seeps have resulted in the contamination of soil and groundwater resources. In the eastern Dahomey basin, observed seeping bitumen is believed to be sourced from offshore source rocks from where they are formed as conventional oil (Coker et al., 1983; Ekweozor & Nwachukwu, 1989). Ekweozor and Nwachukwu (1989) suggested that the biodegradation of original oil migrating up-dip and northward in the direction of the transition margin from the offshore area resulted in the formation of the heavy oil. This statement on microbial degradation of conventional oil to form bitumen was corroborated by chemical degradation experiments, which indicated the presence of asphaltenes in bitumen samples as a by-product of the biodegradation of conventional hydrocarbon and the destruction of its lighter components (Ekweozor & Nwachukwu, 1989). The idea that the seeping bitumen in the eastern flank of the Dahomey basin is not generated in place was supported by the occurrence of bitumen outcrop in the up-dip (onshore) flanks of the basin, as evidence of oil lost from down-dip (offshore) structures by the basin tilting as in a marginal sag basin system (Kingston et al., 1983).

#### Occurrence and composition

The occurrence and distribution of bitumen as seeps have been discussed using geological and geophysical indices within the Nigerian sector of the Dahomey basin (Akinmosin et al., 2011; Ako et al., 1983; Eruteya et al., 2021; Ogunlana et al., 2019; Omosanya et al., 2012). The study of these indices has proven efficient in creating a knowledge suit on the mode of occurrence and distribution of bitumen seeps within the eastern flank of the Dahomey basin. Akinmosin et al. (2011) indicated that structural features such as normal faults in host sedimentary rocks serving as conduits for hydrocarbon migration might be responsible for the formation and consequent near-surface distribution of bitumen seeps. Also, the high porosity of the recent sand units overlying the bitumen-bearing Cretaceous sediments have been suspected of contributing to the formation of seeps, particularly within the eastern flank of the Dahomey basin (Akinmosin et al., 2019). Previous studies focused on the origin, mode of occurrence, and distribution of bitumen within the Nigerian sector of the Dahomey basin has been geared toward the understanding of the economic viability of bitumen exploitation in Nigeria (Akinmosin et al., 2019; Akinsulore & Akinsulore, 2021; Fagbote & Olanipekun, 2010; Ministry of Mines & Steel Development, 2010). The application of geophysical techniques such as electrical resistivity measurements has been used to delineate the occurrence and spatial distribution of known bitumen deposits (Ako et al., 1983; Anukwu et al., 2014; Danielsen et al., 2007). Delineation of bitumen seep zones was made possible due to the contrast in the electrical resistivity property of bitumen and the host sedimentary units, with bitumen generally reported to have higher electrical resistivity above 1000 Ω-m (Odunaike et al., 2010; Omosanya et al., 2012). Understanding the compositional properties of the Nigerian bitumen has led to studies describing both the chemical and physical properties of the Nigerian bitumen to elucidate its economic viability (Adeyemi et al., 2013; Akinmosin et al., 2009; Emmanuel & Ajibade, 2014; Ogiriki et al., 2018). Akinmosin et al. (2019), while studying the viability of exploiting the Nigerian bitumen using the steam-assisted gravity method described the bituminous sediments from core samples obtained from wells within the eastern flank of Dahomey to be generally fine-grained and moderate to well sorted, and the grains are angular to subangular with Porosity ranging from 15.5 to 33.6 ɸ with an average permeability of 4800 mD. A petrographic study using scanning electron microscopy and X-ray diffractometry showed quartz as the dominant mineral component of the bituminous sediments, with subordinate feldspar and other accessory minerals (Akinmosin et al., 2019). The presence of the heavy mineral suite of zircon, tourmaline, and rutile with a ZTR index of 44.7- 61.29 has been reported from bitumen seeping within the Nigerian sector of the Dahomey Basin, with the accumulation of the minerals resulting from the mechanical processes of sedimentation (Akintola et al., 2013). Emmanuel and Ajibade (2014) studied 14 bitumen samples across the Nigerian bitumen belt and determined the elemental composition of the bitumen to consist of carbon, hydrogen, oxygen, nitrogen, and phosphorus in the proportion of 80%, 8%, 4.5%, 4.1%, and 3.9%, respectively, with the nitrogen and phosphorus content an index of biodegradation.

#### Environmental impact

The environmental impact of bitumen seeps in the Nigerian sector of the Dahomey basin has been the subject of recent studies, with the advent of calls for global environmental sustainability and the quest for cleaner exploration and exploitation of hydrocarbon resources. The cases of contaminant fallouts associated with known bitumen exploitation hubs around the globe (Carvalho et al., 2019; Harkness et al., 2018; Roy et al., 2016; Stasik et al., 2015; Sun et al., 2017) have prompted studies on the environmental impact of bitumen exploration in the Nigerian bitumen belt (Adedosu et al., 2021; Asubiojo & Adebiyi, 2011, 2014). These studies have been able to ascertain the presence, type, and concentration of bitumen contaminants in the soil and water resources within the eastern flank of the Dahomey basin. The analysis of soil and water samples from bitumen rich Agbabu area of southwestern Nigeria using the gas chromatography–flame ionization detection (GC-FID) technique indicated the presence of n-alkanes introduced as a result of bitumen impact on the environment (Olajire et al., 2007). The reported concentration of polycyclic aromatic hydrocarbon (PAH) in some sediment samples from bitumen seep zones within the Nigerian bitumen belt was found to be above recommended environmental safety limit (Fagbote & Olanipekun, 2010), this is in tandem with reports of PAHs contamination in regions surrounding bitumen deposits and mining site in Canada (Timoney & Lee, 2009), the PAH observed in porewater samples obtained around the coastal region of the Nigerian sector of the Dahomey basin during dry and wet seasons were dominated by phenanthrene and anthracene (Ololade et al., 2012) which are toxic to plants and animals. Also, benzo (e) pyrene and indenol (1, 2, 3 – cd) pyrene, which are known carcinogens, have been reported in surface water found around bitumen seeps in Irele local government area of southwestern Nigeria, with concentrations of 14.68 ppb and 5.62 ppb respectively (Itodo et al., 2018). The reported concentration is above the maximum allowable concentrations (MACs) as established by United States Agency for Toxic Substances and Disease Registry, which places the safe threshold concentration for both benzo (e) pyrene and indenol (1, 2, 3 – cd) pyrene in the environment at 5.0 ppb (Itodo et al., 2018). The PAHs identified as bitumen-sourced contaminants within the Nigerian sector of the Dahomey basin are listed among the environmental protection agency’s (EPA) 16 priority PAHs of environmental toxicity importance (National Academies of Sciences and Engineering, 2016). Furthermore, heavy metals such as vanadium, chromium, manganese, copper, and zinc, found in association with bitumen deposits, have been reported as groundwater contaminants within the bitumen seep zones of the Nigerian sector of the Dahomey basin (Asubiojo & Adebiyi, 2014). Akinmosin et al. (2009) performed a Gamma-ray spectrometric analysis on air-dried bitumen-contaminated sands obtained within the Dahomey basin to ascertain the propensity of the bitumen-impregnated sand to act as a source of environmental radiogenic contaminant. The observed radiogenic composition of the bituminous sand at 0.446 mSv year^−1^ was considered lower than the threshold where it can constitute an environmental hazard (Akinmosin et al., 2009); however, the presence of radioactivity as a result of the bitumen deposit is a cause for alarm.

#### Mechanism and fate of bitumen contaminants in the environment

Although the presence, nature, and concentration of bitumen-sourced contaminants in soil and groundwater resources from areas of the environment currently affected by in-situ bitumen interactions within the eastern Dahomey basin of southwestern Nigeria have been discussed (Ayandiran et al., 2018; Ojuri et al., 2010; Olajire et al., 2007), the mechanism of interaction of bitumen contaminants with the environment as well as the fate of the contaminants within the soil and groundwater resources has received very little research attention. An understanding of the mechanism of interaction and fate of the various bitumen contaminants is essential to comprehending the distribution, persistence, and re-emergence of the contaminants in the environment. For instance, Ololade et al. (2012) discussed that the presence of organic matter and fines in river sediments directly influences the rate of PAH partitioning into river water and thus affects the fate of the PAH contaminants in the river catchment. However, the authors failed to quantify the rate of partitioning nor describe active processes leading to the partitioning product of bitumen in the environment. There exist a significant gap in knowledge on the processes undergone by bitumen contaminants in the environment within the Nigerian sector of the Dahomey basin. Such processes or mechanism of interaction, as highlighted in previous sections of this review, determines the product and the overall toxicity of bitumen, sourced contaminant in the environment (National Academies of Sciences and Engineering, 2016). The fate of bitumen contaminants is governed by the sorption and desorption mechanism, which controls the rate of retention or attenuation and propagation or distribution of bitumen contaminants in the environment (Brown et al., 2017). Bitumen components released into the environment are either sorbed into sediments which attenuates the distribution of the bitumen contaminants to other parts of the environment, or the bitumen is desorbed into water bodies from whence it is transported and redistributed in the environment (Brown et al., 2017; National Academies of Sciences and Engineering, 2016). Saturated hydrocarbons such as n-alkanes, polycyclic aromatic hydrocarbons such as pyrene, and heavy metals have been reported as soil and groundwater resources contaminants sourced from seeping bitumen within the Nigerian sector of the Dahomey basin (Asubiojo & Adebiyi, 2014; Fagbote & Olanipekun, 2013; Olajire et al., 2007; Ololade et al., 2012), however, the processes controlling the interaction of these contaminants and their fate in the environment is not fully understood.

### Assessment of in situ bitumen seeps contamination

The impact of in situ bitumen seeps contaminants on soil and groundwater resources is best studied using a multidisciplinary approach involving but not limited to the application of geological, geophysical, geochemical, and hydrological techniques aimed at understanding the spatial distribution of seeping bitumen as controlled by structural and stratigraphic heterogeneities within the shallow subsurface (Akinmosin et al., 2011). Also, the combination of GIS and geological mapping with geophysical surveying techniques has been shown to aid decisions on managing bitumen contamination of soil and water resources in an area (Bauman et al., 2000; Ogunlana et al., 2019). Because bitumen components exist in either residual, dissolved, or volatile phases as DNAPL in the environment (Villaume, 1985), they show an affinity for the different phases based on their physical and chemical properties, with their relative abundance in terms of concentration in the different phases’ indicative of their phase preference. Volatile components exist as gases in unsaturated pore spaces or are lost through evaporation (Fingas & Fieldhouse, 2009), soluble components dissolve in ground and surface water thus changing both the chemical and physical properties of the ground or surface water. Non-volatile and insoluble residual components of bitumen are partitioned into those absorbed into soil and aquifer materials, thereby adhering to the sediments within the capillary zones (Huling & Weaver, 1991).

Geophysical surveys such as electrical resistivity measurement of a study site can delineate the location of residual bitumen phases within the saturated and capillary zones (Bauman et al., 2000; Eruteya et al., 2021; Yang et al., 2021). Furthermore, the geochemical analysis of soil and water samples from a test area can inform on the concentration of each bitumen compound existing in all 3 possible phases in the environment. The geochemical result can then be used to model the partitioning, distribution, and fate of bitumen contaminants in soil and groundwater resources (Ryerson et al., 2012). Dissolved bitumen components in the environment are transported or distributed through ground and surface water systems. In order to understand the fate of these dissolved components, characterizing aquifer hydraulic properties and groundwater flow at a DNAPL (Bitumen) contaminated site is necessary. The characterization assists in determining parameters such as the porosity of the aquifer, groundwater flow velocity, hydraulic conductivity, and the hydraulic gradient, which are essential in delineating how the groundwater flow system affects the fate of DNAPL contamination (Rivett & Feenstra, 2005; Sims et al., 1991). Based on the fact that the relationship between geophysical techniques and hydraulic parameters is often indirect and difficult to estimate (Noorellimia et al., 2019), conventional hydrogeological techniques are relied upon to obtain information on the hydraulic parameters of a test area. New or existing groundwater wells with adjoining surface water bodies serving as a hydrogeological boundary are employed for pumping or tracer tests to determine the aquifer's hydraulic properties responsible for DNAPL distribution, transportation, and fate in groundwater (Seyf-Laye et al., 2012).

The integration of results from a multidisciplinary approach through the application of the various techniques highlighted above using empirical principles such as the fugacity model level I and level II (Pollard et al., 2008) is used to predict the fate of bitumen-sourced contaminants in the environment. Level I and II fugacity models were developed to include all phases of DNAPL contaminants within the environment, i.e., air, water, sediment, and non-aqueous phase liquid which represent the source term and can be estimated from the determination of the total petroleum hydrocarbon (Pollard et al., 2008). Level I fugacity (Nieman, 2003) can be used to examine the general partitioning behavior of a DNAPL source such as bitumen seep in the environment, while the level II fugacity model shows the rate or extent of degradation and prevailing degradation mechanisms (Pollard et al., 2008; Sheehan & Kukor, 2020).

### Toxicity or adverse effect of unattended in situ bitumen contaminations

Bitumen seeping into the environment can negatively affect local flora and fauna and cause severe loss of vegetation in the area affected. The presence of adsorbed residual bitumen components results in water repellency which in turn will create a less-than-ideal environment for plant growth by reducing the rate of wetting and retention of water in soils (Roy et al., 2003; Doerr et al., 2000). Tar crust formation has been observed within the bitumen seep zones of the Dahomey Basin. They are the product of extensive weathering and are known to persist on the surface for decades, resulting in vegetation development difficulty by forming a physical barrier that reduces the ingress of oxygen and limits the initial rooting of plants (Brown et al., 2017).

Human ingestion of chemical compounds from in situ bitumen seep can result in health-related challenges. The pollution of ground and surface water with hydrocarbon contaminants from seeping bitumen has been reported within the Dahomey basin in western Nigeria (Asubiojo & Adebiyi, 2014). The most common way humans are exposed to chemicals from bitumen seeps is through the ingestion of contaminated drinking water and food. Lighter and soluble components of bitumen, such as soluble PAHs (e.g., naphthalenes) and BTEXs are important due to their carcinogenic nature (Sims et al., 1991). Drinking water contamination may persist for some time and may occur well beyond the initial seep zones, depending on the fate and transport of the contaminants. Similarly, contamination of agricultural produce by irrigation water is possible as well as fisheries and other domestic food produced with contaminated water.

## Conclusions

Anthropogenic sources of hydrocarbon contaminations often result from isolated and sometimes accidental discharge or processes. However, in the eastern Dahomey basin of southwestern Nigeria, observed bitumen seeps act as a continuous geogenic source of hydrocarbon contamination of soil and groundwater resources within the coastal areas. As a result of their subsurface release into groundwater, evaporative loss of volatile components is minimized, thus increasing the concentration of lighter bitumen components in groundwater. The contaminated groundwater may serve as the medium for redistributing the bitumen contaminants into soil and surface water bodies. Progressive evaporative loss of dissolved bitumen components as a result of exposure to the atmosphere leaves behind the relatively dense and viscous bitumen which can either be submerged in surface water bodies and becomes a secondary source of pollution or adhere to surface sediments forming tar crusts or patches which prevent vegetation development.

The study of soil and groundwater contamination at bitumen-contaminated sites has been the subject of several scientific researches. Although these studies stem from the anthropogenic contamination resulting from exploration, exploitation, and use of bitumen resources, they have made available state-of-the-art techniques for delineating and understanding the dynamics of bitumen contaminants impact in the environment. The synthesis of state-of-the-art techniques for bitumen contamination studies can be applied at regions where there is contamination through geogenic insitu bitumen seeps. Example of such region being the coastal communities of the Nigerian Flank of the Dahomey basin. Evidence of soil and groundwater resources contamination by insitu bitumen seeps has been observed in this region. However, the mode of occurrence of bitumen seeps as well as the nature and fate of the bitumen contaminants in soil and groundwater resources are poorly understood.

The impact and toxicity of hydrocarbon compounds released into soil and groundwater from in-situ bitumen seeps have not received much attention within the coastal communities of the Dahomey basin southwestern Nigeria. Studies within the basin are focused on the anthropogenic sources of bitumen contaminants in the environment along the known bitumen belts at the northmost part of the basin. However, there is a knowledge gap in understanding the environmental effect of in situ bitumen seeps contaminants released into soil and groundwater resources. There is a need to understand the type of seep environment while considering the processes of the bitumen contaminants’ entry into such environments. Also, the geological and sometimes hydrogeological factors controlling the fate and transport of bitumen-sourced contaminants in the environment must be described. This is essential to mitigate the health and socioeconomic consequences of bitumen seep related contamination of soil and groundwater resources in this part Nigeria.

## Supplementary material

The list of articles consulted for this review can be accessed via; JEKAYINFA, Solomon (2022), “A review of the occurrence, distribution and impact of bitumen seeps on soil and groundwater in parts of southwestern, Nigeria 2022”, Mendeley Data, V1, 10.17632/t27pdtbdnc.1

## Data Availability

Data is not available.

## Code availability

None available.
